# Assessment of upright immobilization methods for abdominal and head-and-neck cancer treatments in a carbon ion radiotherapy setting

**DOI:** 10.1016/j.tipsro.2025.100356

**Published:** 2025-11-22

**Authors:** Maria Varnava, Motohiro Kawashima, Akihiko Matsumura, Yoshiaki Oohashi, Makoto Miyazawa, Junichi Koya, Naoto Yamaguchi, Tomoaki Ogano, Mutsumi Tashiro, Tatsuya Ohno

**Affiliations:** a Gunma University Heavy Ion Medical Center, 3-39-22 Showamachi, Maebashi, Gunma 371-8511 Japan; b Department of Radiology, Gunma University Hospital, 3-39-22 Showamachi, Maebashi, Gunma 371-8511 Japan; c Department of Radiation Oncology, Gunma University Graduate School of Medicine, 3-39-15 Showamachi, Maebashi, Gunma 371-8511 Japan

**Keywords:** Carbon ion radiotherapy, Upright patient positioning, Patient immobilization, Interfractional motion, Intrafractional motion

## Abstract

**Background and Purpose:**

Upright patient positioning is an emerging approach in radiotherapy that may overcome current limitations in carbon ion radiotherapy (CIRT). This study evaluated upright immobilization techniques for abdominal and head-and-neck (HN) cancers in a CIRT setting.

**Materials and Methods:**

Ten volunteers were positioned on the Chair, a demo upright positioning system (Leo Cancer Care, USA). Three setups were evaluated: no immobilization devices, vacuum bags alone (ESFORM; Engineering System Co., Japan), and vacuum bags with a thermoplastic shell (Shellfitter; Kuraray Trading Co., Japan), called the “shell” setup. Interfractional and intrafractional errors were assessed using skin markers drawn on the abdominal and HN areas. Interfractional shifts per direction and Euclidean distances were calculated by comparing reference and repeated images. Intrafractional shifts per direction and Euclidean distances were calculated from images acquired over a 15-min period. Also, volunteers rated their comfort for each setup on a scale of 1–5. Differences in the interfractional and intrafractional motion, and comfort ratings between the three setups were investigated.

**Results:**

Significant differences (*p* < 0.05) in the interfractional Euclidean distances were observed between the three setups in both anatomical areas, while significant differences were observed in more shifts per direction in the abdominal case. The “shell” setup minimized intrafractional distances. No significant differences were observed in the comfort ratings between the setups.

**Conclusion:**

Upright positioning in CIRT appears feasible. Further research is needed to refine immobilization techniques to support clinical implementation of upright positioning in CIRT.

## Highlights

• Vacuum bags with a shell reduce interfractional and intrafractional motion.
• Volunteer physical comfort ratings were similar across immobilization setups.
• Further studies should refine immobilization for upright carbon ion radiotherapy.

## Introduction

Radiation therapy is experiencing a significant resurgence in upright patient positioning. Positioning the patient in an upright position, either seating or standing, has been shown to have several advantages compared to the conventional supine position [1,2]. Reported benefits include larger inspiratory and expiratory lung volumes leading to lower mean lung doses, and unaffected prostate position from changes in the bladder filling [1,[3], [4], [5], [6]]. Evidence on respiratory and internal motion is somewhat contradictory; studies suggest decreased internal lung motion, while diaphragm motion seems to be largely unaffected [3,5]. Besides the dosimetric benefits, patients reported greater comfort in the upright position with good reproducibility, which is particularly beneficial for those unable to tolerate lying supine due to dyspnea, saliva accumulation, or dysphagia [2,7].

Integrating an upright patient positioning system in radiotherapy presents the opportunity to develop fixed-beam treatments featuring patient rotation capabilities, a strategy especially relevant for particle therapy. Currently, most carbon ion radiotherapy (CIRT) institutions combine fixed-beam delivery systems with supine treatment couches, limiting available beam directions [8]. While rotating gantry systems provide flexibility, they require a substantial space and are more expensive [9]. Upright positioning systems could overcome these limitations by enabling patient rotation and reducing infrastructure costs, thus making particle therapy more accessible globally. Moreover, reduced organ motion, favorable anatomical positioning, and optimized beam directions may improve dose distributions, and consequently improve treatment outcomes [1,8,9].

Previous studies have investigated interfractional and intrafractional reproducibility in upright setups for X-ray radiotherapy and particle therapy [7,[10], [11], [12],^13^]. Boisbouvier et al. reported mean interfractional shifts of < 1 mm and intrafractional shifts of ≤ 3 mm in the majority of cases in pelvic radiotherapy using a prototype chair [10]. McCarroll et al. observed 2-mm mean interfractional and < 3-mm mean intrafractional errors in head-and-neck (HN) cancer patients using a constructed treatment chair [7]. A six-degrees-of-freedom chair has also been developed, and its mechanical accuracy and clinical applicability were evaluated [11,14,15]. Mean intrafractional errors for HN cancer patients were < 1 mm. Recently, Feldman et al. evaluated motion in HN proton therapy, reporting mean interfractional shifts between −3.7 ± 3.5 mm and 0.5 ± 6.2 mm on daily CT registrations, and mean intrafractional shifts between −0.31 ± 1.37 mm and 0.59 ± 1.55 mm from kilovoltage imaging [12]. Koromi et al. reported mean interfractional errors ranging from −1.4 mm to −0.5 mm by aligning computed radiographs based on bone structures in HN cancer patients treated with boron neutron capture therapy (BNCT) in a semi-recumbent position [13].

In CIRT, dose distributions are far more sensitive to anatomical changes than in X-ray radiotherapy due to range uncertainties, highlighting the importance of reproducibility [16]. Upright interfractional and intrafractional errors should therefore be equal to or smaller than those reported in X-ray radiotherapy and particle therapy [7,[10], [11], [12],^13^]. Immobilization approaches may need to vary by tumor site and positioning system: pelvic cancer patients have been immobilized with a vacuum bag and a belt, while HN tumors may benefit from shells. Therefore, it is necessary to systematically evaluate upright immobilization techniques for different treatment sites and positioning systems.

The purpose of this study is to evaluate interfractional and intrafractional errors in abdominal and HN sites using three immobilization setups in a CIRT setting: no immobilization, vacuum bags alone, and vacuum bags combined with thermoplastic shells.

## Materials and methods

### Volunteers

The study was approved by our institutional review board (number HS2024-106). Data were collected prospectively by enrolling ten healthy volunteers. All volunteers included in this study were at least 18 years old and gave informed consent prior to data collection. The volunteer characteristics are shown in Table 1.

**Table 1:** Volunteers’ characteristics.

**Number of volunteers**	n = 10
**Gender** Male Female	n = 10 n = 0
**Age (years)** Mean ± standard deviation Range	36.8 ± 9.7 23–53
**Height (cm)** Mean ± standard deviation Range	171.8 ± 2.2 168–175
**Weight (kg)** Mean ± standard deviation Range	73.5 ± 9.7 62–91

### Experimental setup and workflow

To evaluate upright position immobilization setups in CIRT, a demo upright patient positioning system (Leo Cancer Care, USA), referred to as the Chair, was temporarily installed at our institution. The Chair was supplemented with an optical guidance and tracking system (OGTS; Leo Cancer Care, USA) that was developed for patient positioning. The OGTS comprised of three high resolution cameras and an OGTS software. Two cameras were placed orthogonally, frontal and lateral to the Chair, and the third one was placed in an oblique direction between the other two (Supplementary Fig. 1).

For each volunteer, the experiment was conducted over two days. On the first day, immobilization devices, vacuum bag(s) (ESFORM; Engineering System Co., Japan) and a thermoplastic shell (Shellfitter; Kuraray Trading Co., Japan), were created by at least two radiologic technologists. Five technologists participated in the research team, with clinical experience in radiation therapy of 20, 17, 15, 12, and 7 years. For the abdominal setup, the volunteers were asked to remove all clothing from their upper body. A single vacuum bag was used for the abdominal setup, and two vacuum bags were used for the HN setup, one to support the head and one to support the waist and hips. All volunteers were set up on the Chair following the procedure described in Supplementary Fig. 2 by two radiologic technologists, supported by two physicists (with 15 and 4 years of clinical experience). The Chair components were adjusted to improve the stability and comfort for each volunteer, except the backrest which was fixed at 0° due to experimental setup limitations. Supplementary Fig. 3 shows the experimental setup for taking abdominal and HN measurements. To minimize instability of the upper vacuum bag for the HN setup, the arm rest supports were used.

Interfractional and intrafractional motion were evaluated based on shifts of specific points on the volunteers’ skin. Markers were placed on easily identifiable anatomical landmarks before creating the shell, and openings on the shell were created to allow monitoring of these points (Supplementary Fig. 4). Nine markers were placed on the abdominal area and seven on the HN area. Using the OGTS, images were acquired for both anatomical areas for all three setups: with all immobilization devices (“shell” setup), with only vacuum bags (“vacuum” setup), and without any immobilization devices (“none” setup).

On the second day, the volunteer was repositioned on the Chair for the “shell” setup based on the images acquired the prior day using the OGTS, mainly by the two physicists. Markers were drawn on the skin at the center of the shell openings, and reference images from all cameras were acquired using the SVCapture software (SVS-Vistek, Germany). The volunteer was then positioned for the “vacuum” and “none” setups using the OGTS, and reference images were acquired each time using SVCapture. Positioning of each volunteer in the “shell”, “vacuum”, and “none” setups was repeated five times, and images were acquired each time to assess interfractional motion. The fifth time, a 15-min video was recorded in the form of consecutive images using SVCapture for each setup to evaluate intrafractional motion. This process was conducted for both the abdominal and HN cases. During the image acquisition of interfractional measurements for the abdominal case, the breathing was controlled; the volunteer was asked to synchronize exhalation with image capture in order to minimize breathing effects on the marker motion. Similarly, for the HN case, the volunteer was asked to keep their mouth closed in a relaxed position during image capture.

In total, five interfractional images and fifteen intrafractional images were acquired for each of the abdominal and HN areas, excluding the reference images. The intrafractional images were extracted at 1-min intervals from the 15-min video.

### Marker coordinates in 3D space

The position of each marker was determined in 3D space using OpenCV-based software [17]. The software was based on the pinhole camera model and was developed by the National Institutes for Quantum Science and Technology (QST). The software gives the coordinates of a marker in the left-right (LR), superior-inferior (SI), and anterior-posterior (AP) directions. A point laterally centered on the surface of the Chair was defined as the (0,0,0) coordinate. The software requires pixel positions from two cameras to calculate the marker coordinates. This implies that the marker has to be visible from at least two cameras to be able to generate coordinates.

Pixel positions for all markers were obtained from the frontal, oblique, and lateral images using ImageJ (National Institutes of Health, USA). For each marker, we utilized the pixel positions from the cameras with the best visibility. For the abdominal case, coordinates for the markers #4, #5, #8, and #9 were estimated using the pixel positions from the lateral and oblique cameras. For the HN case, the markers #3 and #6 were estimated using the pixel positions from the lateral and oblique cameras. The remaining markers for the abdominal and HN cases were estimated using the pixel positions from the frontal and oblique cameras.

### Interfractional and intrafractional motion

The shift of a marker in the LR/SI/AP direction was defined as the deviation in the LR/SI/AP direction from the reference image. The Euclidean distance a marker moved was defined as:

$$\mathrm{distance}=\sqrt{{\Delta x}^{2}+{\Delta y}^{2}+{\Delta z}^{2}}$$

where Δx, Δy, and Δz are the shifts in the LR, SI, and AP directions, respectively.

### Physical comfort evaluation of the upright positioning experience

Volunteers were asked to rate their physical comfort in the upright position for each setup between 1 and 5; 1 for very painful, 2 for slightly painful, 3 for neither painful nor comfortable, 4 for comfortable, and 5 for very comfortable.

### Statistical analysis

Statistical analyses were performed using IBM SPSS Statistics (IBM Corp, USA). Data are presented as the mean ± standard deviation. The shifts and distances were calculated by considering all markers for all volunteers. Shapiro-Wilk test was used to investigate the normality of the data. The Friedman test was used to investigate the differences in the shifts per direction, Euclidean distances, and volunteer comfort levels between the three setups for both the abdominal and HN cases. For the intrafractional motion analysis, the mean of the shifts in each direction was evaluated at 1-min intervals up to the 15th min after recording.

## Results

**Fig. 1:**
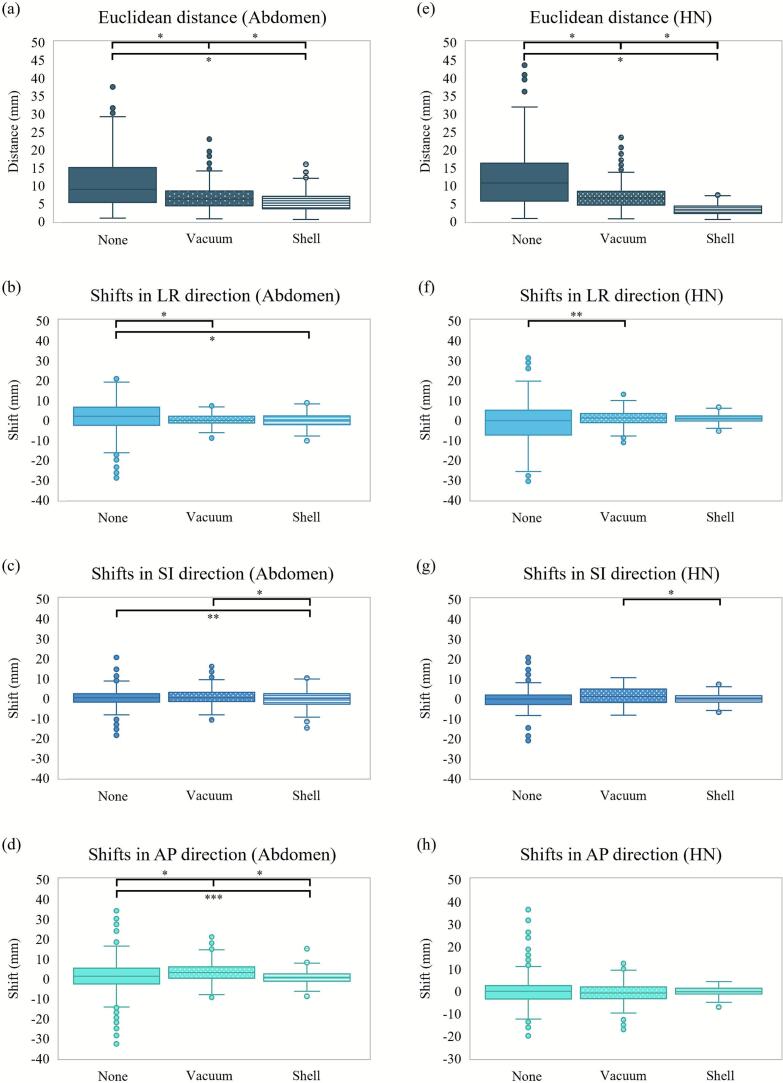
Interfractional motion in the abdominal and head-and-neck (HN) cases. (a) Euclidean distance and shifts in the (b) left-right (LR), (c) superior-inferior (SI), and (d) anterior-posterior (AP) directions for the abdominal case. (e) Euclidean distance and shifts in the (f) LR, (g) SI, and (h) AP directions for the HN case. Each box plot represents the first quartile, median, and third quartile of the data. The whiskers correspond to ± 1.5 × interquartile range. (*: *p* ≤ 0.001, **: *p* ≤ 0.01, ***: *p* < 0.05).

Interfractional motion analysis showed significant differences in all Euclidean distances between the three setups for both the abdominal and HN cases (Fig. 1, Fig. 1). There were significant differences in almost all shifts per direction for the abdominal case, except in the LR direction between the “none” and “vacuum” setups and in the SI direction between the “vacuum” and “shell” setups (Fig. 1, Fig. 1b–d). On the other hand, for the HN case, significant differences were only found in the LR direction between the “none” and “vacuum” setups and in the SI direction between the “vacuum” and “shell” setups (Fig. 1, Fig. 1f–h). Table 2 summarizes the mean values of the interfractional shifts in each direction and distances. Furthermore, examining the motion of each marker showed that immobilization devices generally resulted in reduced interfractional motion for both the abdominal and HN cases (Supplementary Fig. 5).

**Table 2:** Mean and standard deviation (range) values of the interfractional motion in the left-right (LR), superior-inferior (SI), and anterior-posterior (AP) directions and the Euclidean distance for the abdominal and head-and-neck cases. All markers of all patients are considered.

**Setup**	**LR-direction shift (mm)**	**SI-direction shift (mm)**	**AP-direction shift (mm)**	**Euclidean distance** **(mm)**
**Abdominal case**				
**“None”**	0.8 ± 9.2 (−29.1–20.8)	−0.1 ± 4.6 (−18.6–20.5)	0.7 ± 8.6 (–32.8–35.2)	11.6 ± 7.6 (0.9–38.1)
**“Vacuum”**	0.3 ± 2.8 (−9.0–8.6)	0.7 ± 4.2 (−10.8–16.0)	3.2 ± 4.6 (−9.4–21.0)	6.7 ± 3.4 (0.7–22.9)
**“Shell”**	0.0 ± 3.1 (−10.3–9.9)	−0.4 ± 4.1 (−14.8–12.1)	0.7 ± 3.2 (−8.9–15.1)	5.5 ± 2.6 (0.5–15.9)
**HN case**				
**“None”**	−1.2 ± 11.2 (−30.7–31.2)	−0.3 ± 5.8 (−21.1–21.7)	0.3 ± 7.6 (−19.9–36.4)	11.7 ± 8.2 (0.2–44.6)
**“Vacuum”**	1.1 ± 4.0 (−11.3–13.7)	1.3 ± 4.4 (−8.4–10.5)	−0.9 ± 4.7 (−17.1–12.4)	5.4 ± 2.9 (0.3–16.0)
**“Shell”**	0.8 ± 2.0 (−5.5–6.5)	0.0 ± 2.5 (−6.8–7.4)	−0.2 ± 1.9 (−7.1–4.2)	2.9 ± 1.6 (0.1–7.7)

**Fig. 2:**
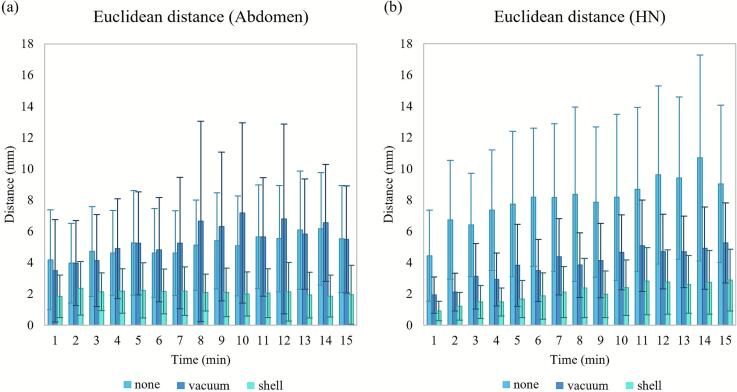
Variation of the intrafractional Euclidean distance over a 15-min time period for the (a) abdominal and (b) head-and-neck (HN) cases. Error bars represent ± standard deviation.

**Fig. 3:**
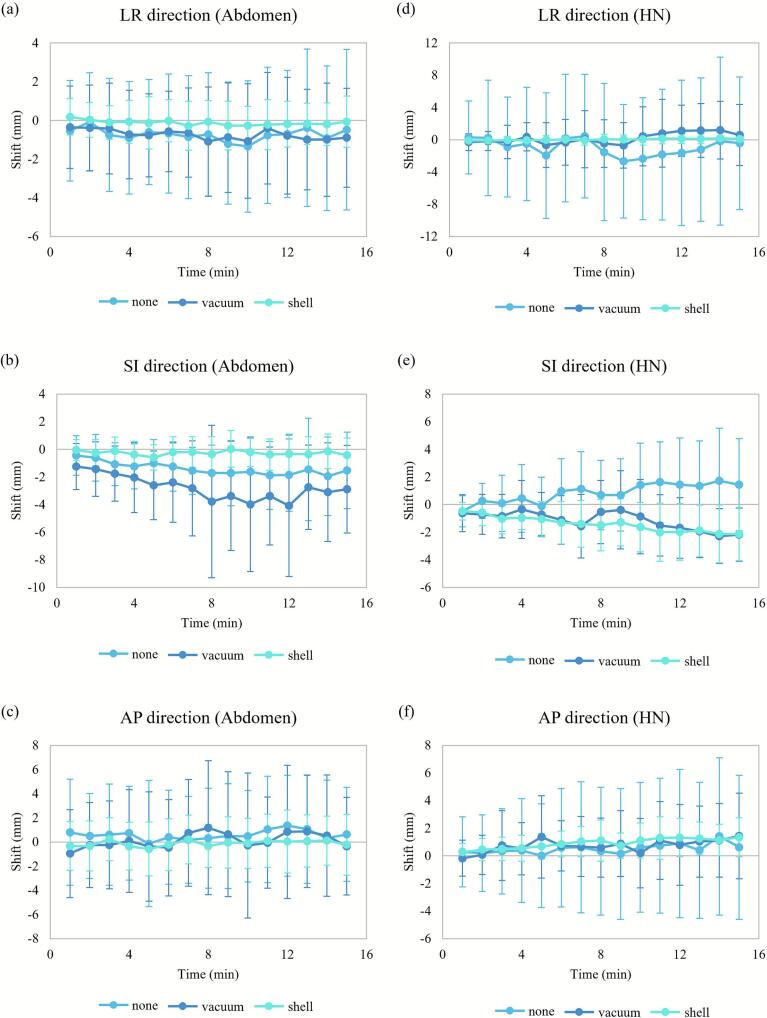
Intrafractional mean shifts per direction over a 15-min time period for the abdominal and head-and-neck (HN) cases. Mean values were calculated for every minute using the markers from all volunteers. Intrafractional shifts are shown for the (a) left-right (LR), (b) superior-inferior (SI), and (c) anterior-posterior (AP) directions in the abdominal case, and for the (d) LR, (e) SI, and (f) AP directions in the HN case. Error bars represent ± standard deviation.

Fig. 2 shows the intrafractional distance variation over a 15-min time period, and Table 3 summarizes the mean shifts per direction and distances for every minute of the 15-min time period. The abdominal “none” and “vacuum” setups had similar distance values per minute, while the “shell” setup had the smallest distance. The HN “none” setup had the largest distance, whereas the “shell” setup had the smallest one. Fig. 3 shows how shifts varied over time in each direction for the three setups. In both the abdominal and HN cases, the “shell” setup had the smallest shift variations in all directions. In the abdominal case, similar trends were observed for the mean shifts in the LR and AP directions for all setups. In the HN case, similar trends were observed for the mean shifts in the LR and AP directions for all setups, and in the SI direction for the “vacuum” and “shell” setups.

**Table 3:** Mean and standard deviation (range) values of the intrafractional motion in the left-right (LR), superior-inferior (SI), and anterior-posterior (AP) directions and the Euclidean distance for the abdominal and head-and-neck cases. The shifts of all markers of all patients for every minute over the 15-min period are considered.

**Setup**	**LR-direction shift (mm)**	**SI-direction shift (mm)**	**AP-direction shift (mm)**	**Euclidean distance** **(mm)**
**Abdominal case**				
**“None”**	−0.7 ± 3.3 (−13.2–9.0)	−1.4 ± 2.3 (−10.6–11.1)	0.6 ± 4.2 (−13.4–18.3)	5.1 ± 3.2 (0.2–18.7)
**“Vacuum”**	−0.7 ± 2.6 (−12.3–6.6)	−2.8 ± 3.6 (−31.4–4.7)	0.1 ± 4.6 (−14.4–25.9)	5.5 ± 4.3 (0.2–32.2)
**“Shell”**	−0.1 ± 1.1 (−4.3–4.5)	−0.3 ± 1.1 (−4.6–3.0)	−0.1 ± 2.1 (−8.2–8.3)	2.2 ± 1.5 (0.1–9.4)
**HN case**				
**“None”**	−0.9 ± 7.8 (−24.4–19.7)	0.8 ± 2.8 (−5.5–11.6)	0.5 ± 4.4 (−12.0–17.4)	8.1 ± 5.0 (0.2–25.3)
**“Vacuum”**	0.2 ± 3.0 (−9.2–12.9)	−1.2 ± 2.1 (−6.5–7.8)	0.7 ± 2.4 (−6.6–11.9)	3.9 ± 2.5 (0.2–13.6)
**“Shell”**	0.0 ± 0.6 (−2.0–1.9)	−1.4 ± 1.7 (−7.2–2.0)	0.9 ± 1.2 (−1.6–5.1)	2.1 ± 1.7 (0.0–7.2)

Comparisons of the physical comfort ratings showed no significant differences (*p* > 0.05) between the setups for both cases. A summary of the ratings is shown in Table 4. Six volunteers gave the same rating for all three setups for the abdominal case, and four volunteers gave the same rating for the HN case. The volunteers that gave different ratings between the setups tended to prefer the “vacuum” setup over the “none” in the abdominal case, while in the HN case, the volunteers tended to prefer the “shell” setup.

**Table 4:** Mean and standard deviation (range) values of the physical comfort rating.

**Setup**	**Rating**
**Abdominal case**	
**“None”**	3.1 ± 0.5 (2–4)
**“Vacuum”**	3.3 ± 0.8 (2–5)
**“Shell”**	3.3 ± 0.8 (2–5)
**HN case**	
**“None”**	3.2 ± 0.6 (2–4)
**“Vacuum”**	3.2 ± 0.4 (3–4)
**“Shell”**	3.5 ± 0.9 (2–5)

## Discussion

We evaluated three immobilization setups for abdominal and HN areas in a CIRT setting. The “shell” setup reduced both interfractional and intrafractional Euclidean distances compared with the other setups in both anatomical regions. The “vacuum” setup reduced interfractional motion compared with the “none” setup, but in the abdominal case did not improve intrafractional motion (Fig. 2a), reflecting the absence of posterior abdominal immobilization and leaving respiratory motion unaffected. Thus, combining vacuum bags and thermoplastic shells appears necessary for optimal setup accuracy and reproducibility. Across both areas, intrafractional shifts showed similar directional patterns, but the “shell” setup consistently yielded smaller standard deviations, underscoring its effectiveness in minimizing motion. However, directional shift analysis showed that, unlike in the abdominal case, immobilization devices did not significantly reduce interfractional shifts in most directions for HN. This suggests that the construction and fixation of both the HN vacuum bags and the thermoplastic shell were suboptimal, highlighting the need for improvements in their stability and construction.

Our results are comparable with previous upright studies in X-ray radiotherapy [7,10,11]. Boisbouvier et al. used the same upright positioning system as our study and reported interfractional shifts of −0.5 ± 2.5 mm, −0.9 ± 2.7 mm, and −0.4 ± 1.3 mm in the LR, SI, and AP directions, respectively, for pelvic radiotherapy [10]. These agree with our results for the HN “shell” setup, which is not strongly affected by respiration. Also, most intrafractional shifts were within 3 mm, consistent with our observations in the LR and AP directions. Unlike Boisbouvier at al. who calculated shifts by manually aligning images using the OGTS, our study evaluated 3D skin-marker motion, which may provide a more accurate representation of displacement. However, both studies rely on the external body anatomy to calculate motion, which might not accurately reflect internal motion. Further research integrating imaging would be necessary to confirm whether the reproducibility of external anatomy corresponds to that of internal targets. McCarroll et al. reported < 2 mm mean interfractional and < 3 mm intrafractional errors for six HN subregions by registering kilovoltage images in upright radiotherapy using a prototype chair [7]. This is consistent with our results for the HN “shell” setup; mean interfractional and intrafractional shifts/distance were within 1/3 mm and 2/3 mm, respectively. Sun et al. reported intrafractional errors of −0.08 ± 0.56 mm, 0.71 ± 1.12 mm, and −0.52 ± 0.84 mm in the LR, SI, and AP directions, respectively for HN cancer patients with a six-degree-of-freedom chair, which also agrees with our results [11].

Interfractional and intrafractional errors for upright radiotherapy have also been reported in particle therapy [12,13,18]. Feldman et al. observed mean interfractional shifts between −3.7 ± 3.5 mm and 0.5 ± 6.2 mm from daily CT registrations, and mean intrafractional shifts between −0.31 ± 1.37 mm and 0.59 ± 1.55 mm from kilovoltage imaging between fractions in upright HN proton therapy [12]. These values are comparable to our “shell” setup shifts for both anatomical sites. Koromi et al. evaluated setup reproducibility of HN cancer patients treated with BNCT in a semi-recumbent position, imitating the sitting position [13]. Interfractional errors were assessed by aligning computed radiographs acquired before planning CT and before treatment based on bone structures. Mean interfractional errors of −0.5 ± 1.3 mm, −0.5 ± 2.2 mm, and −1.4 ± 1.5 mm in the LR, SI, and AP directions, respectively, were observed, which are consistent with our results for the HN “shell” setup. Currently, there are no upright CIRT studies reporting patient reproducibility. However, a supine CIRT study for prostate cancer monitored interfractional and intrafractional motion using an implanted marker placed at the center of the planning target volume with a fluoroscopy imaging system [18]. The mean interfractional and intrafractional Euclidean distances observed were 1.68 ± 1.11 mm, and 0.76 ± 0.54 mm, respectively. In our study, larger distances were observed for the HN “shell” setup when monitoring skin markers.

In CIRT, setup errors and anatomical changes can significantly impact the range of carbon ions, directly influencing dose distributions to the target and normal tissues [[19], [20], [21], [22]]. This study focused on interfractional and intrafractional errors caused by patient setup. The mean interfractional/intrafractional Euclidean distances for the “shell” setup for the abdominal and HN cases were 5.5 ± 2.6 mm/2.2 ± 1.5 mm and 2.9 ± 1.6 mm/2.1 ± 1.7 mm, respectively. Image guidance can mitigate interfractional errors. Our intrafractional errors, though small, are clinically significant. Such errors can be mitigated by adjusting setup margins and improving immobilization techniques.

Physical comfort in the upright position has been evaluated in previous studies. Using a 1–5 rating scale (with 5 indicating maximum comfort), Boisbouvier et al. reported a mean overall comfort score of 4.1 for pelvic cancer patients, and Feldman et al. found a mean overall comfort score of 3.75 for four HN patients [10,12]. McCarroll et al. observed a mean overall comfort level of 4.6 for HN patients positioned leaning forward on a chair, based on a 0–5 scale [7]. In our study, the mean comfort ratings were 3.3 ± 0.8 and 3.5 ± 0.9 for the abdominal and HN “shell” setups, respectively. The comfort level of our HN “shell” setup is consistent with that reported by Feldman et al. whose patient positioning was similar despite differences in chair design [12]. Overall, these findings suggest that upright positioning is generally tolerable and comfortable for most participants.

Feedback from volunteers indicated the need for improved immobilization. In the abdominal setup, discomfort was reported in the arms and elbows due to the arm rest, while in the HN “shell” setup, discomfort was mainly attributed to the Chair backrest being fixed at 0°. Tilting the backrest at 5° or 10° could improve comfort, but this was not feasible in our study because facial markers became invisible to the cameras. Another design limitation was the wide backrest, which made tailoring the shells difficult. A narrower backrest could improve fitting and reduce deviations. This study was also based on deviations of skin points, which introduced potential skin displacement during shell attachment. Although care was taken to minimize this, such displacements only affect the interfractional error, which in clinical practice can be corrected through image guidance. Additional limitations include the short experimental timeframe and setup, which restricted investigation of more appropriate techniques. Due to lack of experience with upright positioning and limited time, the radiologic technologists used the same immobilization methods to those employed in supine CIRT clinical setups. The study also employed a demo system that differed from the commercial version in terms of chair component stability and ground fixation, meaning our results may not fully represent the commercial product’s performance. Chair rotation was also not possible with the demo system. For treatments requiring multiple beam angles within a treatment session, additional errors may arise when rotating the commercial Chair from one beam angle to another. However, such errors can be compensated through image guidance before beam delivery. Furthermore, the lack of integrated CT imaging, prevented us from determining whether the observed motion accurately reflects interfractional and intrafractional shifts of internal anatomical landmarks, complicating comparisons with studies that evaluated internal landmarks rather than skin surface. Moreover, our sample was small and limited to healthy, relatively young male volunteers. The abdominal marker placement and the lack of female radiologic technologists at the time of the study limited female enrolment. Therefore, our sample may not accurately reflect patient populations, who are usually older and may experience cancer-related symptoms, possibly facing greater discomfort in upright positioning and potentially leading to larger interfractional and intrafractional errors.

## Conclusion

Despite limitations, this study demonstrates the feasibility of upright positioning in CIRT. Immobilization using vacuum bags and thermoplastic shells effectively reduced both interfractional and intrafractional motion in the evaluated anatomical sites, with results consistent with previous upright studies in X-ray radiotherapy and particle therapy. The observed intrafractional errors are clinically relevant for CIRT but could likely be mitigated by adjusting target setup margins for early implementation of upright CIRT. Further optimization of immobilization techniques to improve comfort and reproducibility, as well as elucidation of correlations between skin motion and internal anatomical motion, will be necessary to fully realize the clinical potential of upright positioning in CIRT.

## Declaration of competing interest

The authors declare that they have no known competing financial interests or personal relationships that could have appeared to influence the work reported in this paper.

## References

[1] Volz L., Sheng Y., Durante M., Graeff C. (2022). Considerations for Upright particle therapy patient positioning and associated image guidance. Front Oncol.

[2] Rahim S., Korte J., Hardcastle N., Hegarty S., Kron T., Everitt S. (2020). Upright radiation therapy-a historical reflection and opportunities for future applications. Front Oncol.

[3] Yang J., Chu D., Dong L., Court L.E. (2014). Advantages of simulating thoracic cancer patients in an upright position. Pract Radiat Oncol.

[4] Yamada Y., Yamada M., Chubachi S., Yokoyama Y., Matsuoka S., Tanabe A. (2020). Comparison of inspiratory and expiratory lung and lobe volumes among supine, standing, and sitting positions using conventional and upright CT. Sci Rep.

[5] Marano J., Kissick M.W., Underwood T.S.A., Laub S.J., Lis M., Schreuder A.N. (2023). Relative thoracic changes from supine to upright patient position: a proton collaborative group study. J Appl Clin Med Phys.

[6] Mackie T.R., Towe S., Schreuder N. (2021). Is upright radiotherapy medically and financially better?. AIP Conf Proc.

[7] McCarroll R.E., Beadle B.M., Fullen D., Balter P.A., Followill D.S., Stingo F.C. (2017). Reproducibility of patient setup in the seated treatment position: a novel treatment chair design. J Appl Clin Med Phys.

[8] Miyasaka Y., Lee S.H., Souda H., Kaneko T., Hagiwara Y., Chai H. (2024). Treatment planning comparison of gantry-based and fixed beams for the treatment of liver tumors with carbon ion therapy. In Vivo.

[9] Chinniah S., Deisher A.J., Herman M.G., Johnson J.E., Mahajan A., Foote R.L. (2023). Rotating gantries provide individualized beam arrangements for charged particle therapy. Cancers (Basel).

[10] Boisbouvier S., Boucaud A., Tanguy R., Grégoire V. (2022). Upright patient positioning for pelvic radiotherapy treatments. Tech Innov Patient Support Radiat Oncol.

[11] Sun J., Kong L., Chen Z., You D., Mao J., Guan X. (2021). Clinical implementation of a 6D treatment chair for fixed ion beam lines. Front Oncol.

[12] Feldman J., Pryanichnikov A., Shwartz D., Hillman Y., Wygoda M., Blumenfeld P. (2024). Study of upright patient positioning reproducibility in image-guided proton therapy for head and neck cancers. Radiother Oncol.

[13] Komori S., Hirose K., Sato M., Yamazaki Y., Takeuchi A., Kato R. (2024). Retrospective analysis of treatment-positioning accuracy and dose error in boron neutron capture therapy using a sitting-position treatment system for head and neck cancer. Phys Med.

[14] Sheng Y., Sun J., Wang W., Stuart B., Kong L., Gao J. (2020). Performance of a 6D treatment chair for patient positioning in an upright posture for fixed ion beam lines. Front Oncol.

[15] Zhang X., Hsi W.C., Yang F., Wang Z., Sheng Y., Sun J. (2020). Development of an isocentric rotating chair positioner to treat patients of head and neck cancer at upright seated position with multiple nonplanar fields in a fixed carbon-ion beamline. Med Phys.

[16] Mohamad O., Sishc B.J., Saha J., Pompos A., Rahimi A., Story M.D. (2017). Carbon ion radiotherapy: a review of clinical experiences and preclinical research, with an emphasis on DNA damage/repair. Cancers.

[17] Bradski G. (2000). The opencv library. Dr Dobb's J.

[18] Iwai Y., Mori S., Ishikawa H., Kanematsu N., Matsumoto S., Nakaji T. (2024). Inter-fractional error and intra-fractional motion of prostate and dosimetry comparisons of patient position registrations with versus without fiducial markers during treatment with carbon-ion radiotherapy. Radiol Phys Technol.

[19] Ammazzalorso F., Jelen U., Engenhart-Cabillic R., Schlegel W. (2014). Dosimetric robustness against setup errors in charged particle radiotherapy of skull base tumors. Radiat Oncol.

[20] Fattori G., Riboldi M., Scifoni E., Krämer M., Pella A., Durante M. (2014). Dosimetric effects of residual uncertainties in carbon ion treatment of head chordoma. Radiother Oncol.

[21] Sakama M., Kanematsu N. (2018). An evaluation method of clinical impact with setup, range, and radiosensitivity uncertainties in fractionated carbon-ion therapy. Phys Med Biol.

[22] Li Y., Kubota Y., Okamoto M., Shiba S., Okazaki S., Matsui T. (2021). Adaptive planning based on single beam optimization in passive scattering carbon ion radiotherapy for patients with pancreatic cancer. Radiat Oncol.

