# Supplementary material for: Assessment of upright immobilization methods for abdominal and head-and-neck cancer treatments in a carbon ion radiotherapy setting
Source: Tech Innov Patient Support Radiat Oncol. 2025 Nov 22;36:100356. doi: 10.1016/j.tipsro.2025.100356 (PMC12686792; doi:10.1016/j.tipsro.2025.100356)
Supplement: Supplementary Data 1 [file mmc1.pdf]

Supplementary Figure S1. Diagram of experimental setup.

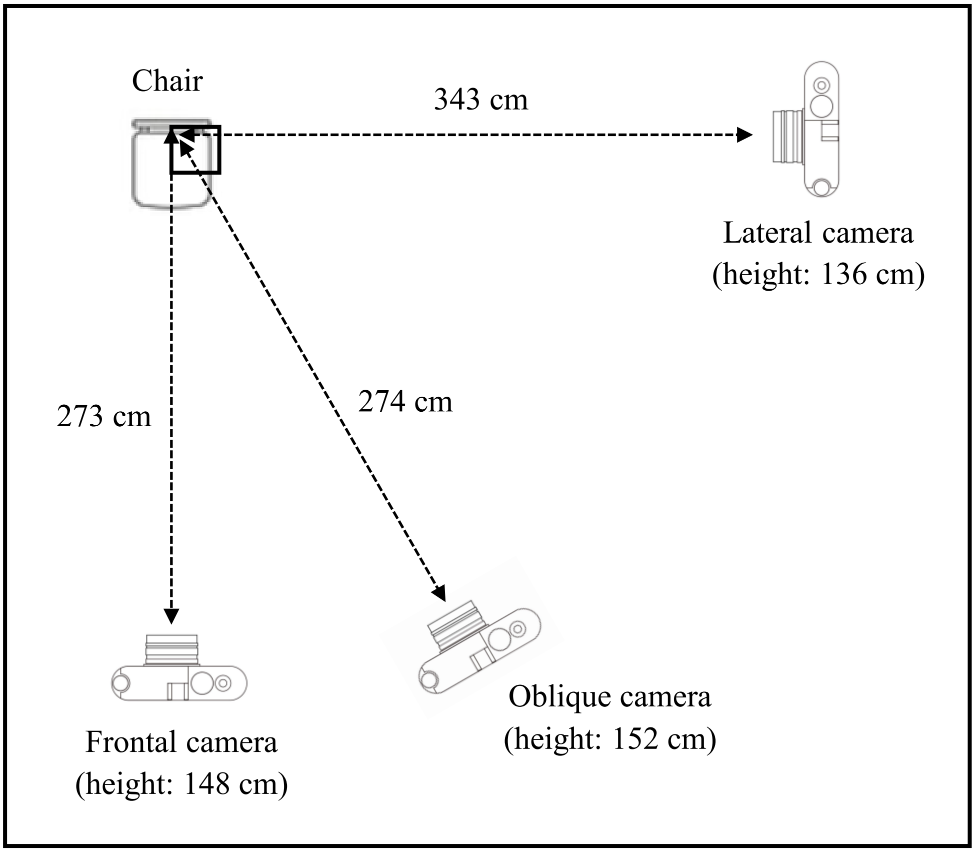

Supplementary Figure S2. The procedure followed to setup the volunteers on the Chair.

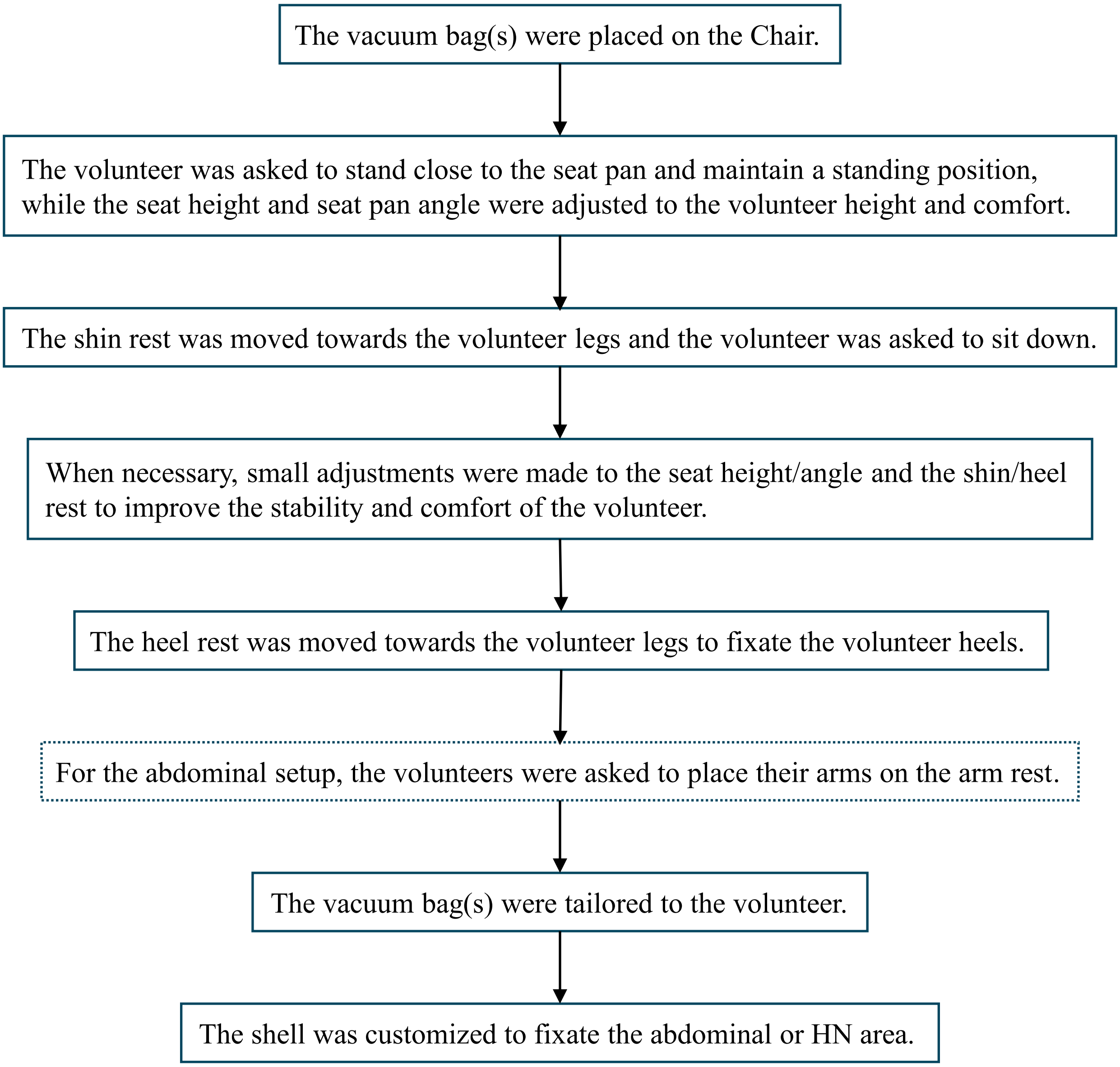

Supplementary Figure S3. Experimental setup. Abdominal setup as viewed from the (a) frontal and (b) lateral cameras. Head-and-neck setup as viewed from the (c) frontal and (d) lateral cameras.

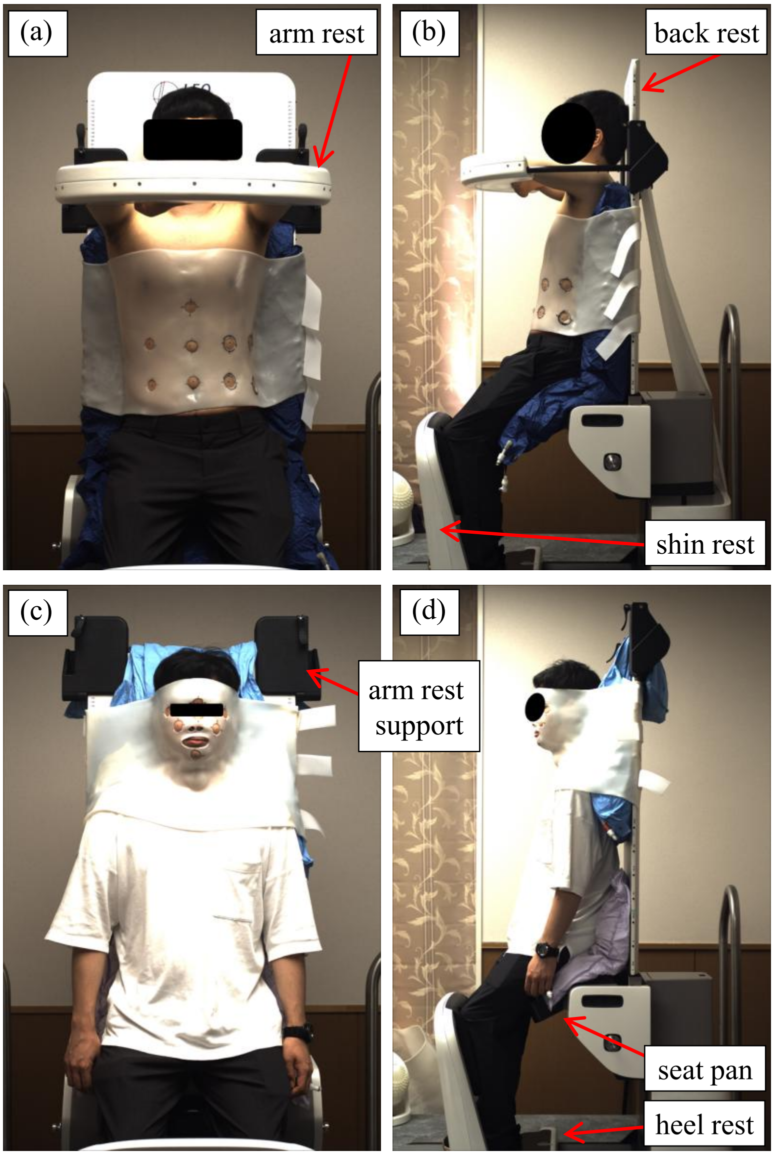

Supplementary Figure S4. Marker positions for the (a), (b) abdominal and (c), (d) head-and-neck cases as viewed from the frontal and lateral cameras.

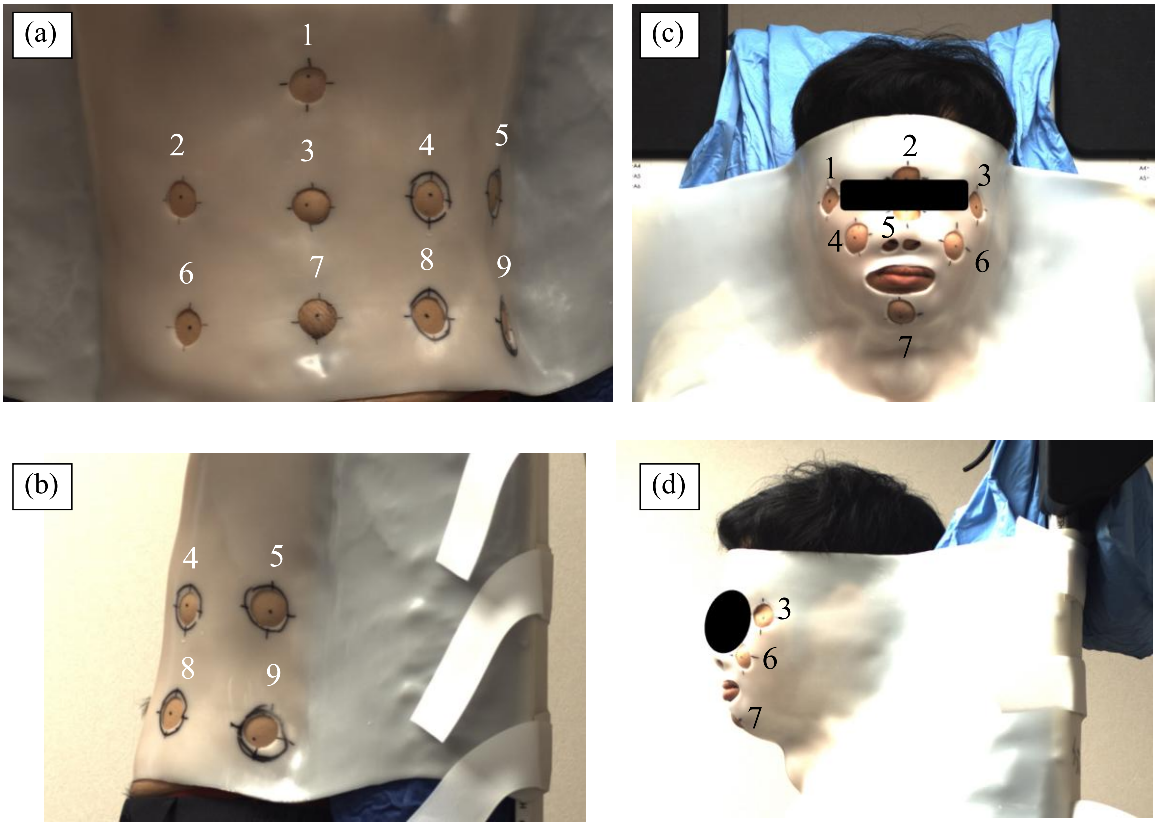

The markers were placed on easily identifiable landmarks.

Abdominal case markers

#1: xiphoid process

#2: 10 cm to the right of #3

#3: in the middle of the #1 and #7 markers

#4: 10 cm to the left of #3

#5: further displaced laterally from #4

#6: 10 cm to the right of #7

#7: 2 cm above the umbilicus

#8: 10 cm to the right of #7

#9: further displaced laterally from #8

Head-and-neck case markers

#1: 1 cm to the right from the outer corner of the right eye

#2: 3 cm above the glabella

#3: 1 cm to the right from the outer corner of the left eye

#4: lowest part of the right zygomatic bone

#5: middle of the nasal dorsum

#6 : lowest part of the left zygomatic bone

#7: 1–2 cm below the lower lip
(depending on the volunteer's face anatomy)

Supplementary Figure S5. Mean interfractional Euclidean distance of each marker for the (a) abdominal and (b) head-and-neck (HN) case. Error bars correspond to ± standard deviation.

(a)

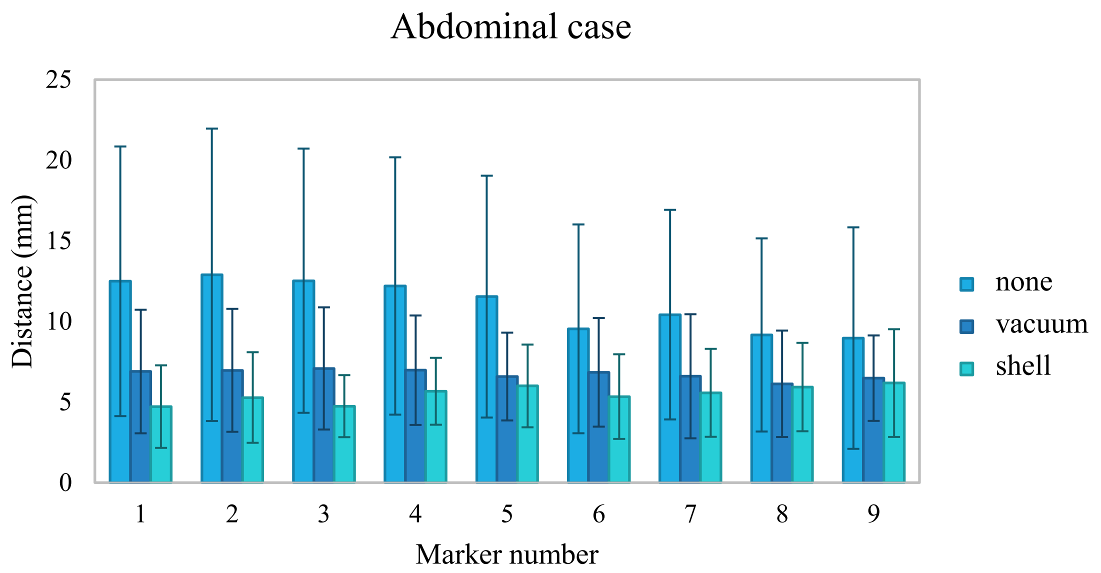

(b)

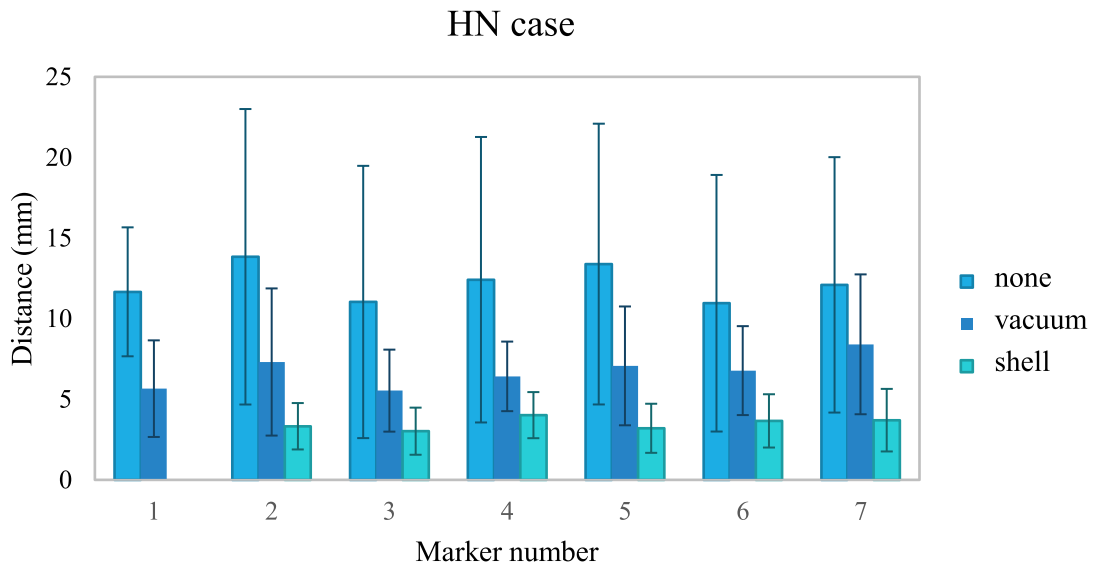

*In the HN case, marker #1 was not visible during the "shell" setup.
